# The Interplay of Magnetic Order with the Electronic Scattering and Crystal‐Field Effects in a Metallic Ferromagnet

**DOI:** 10.1002/advs.202517704

**Published:** 2025-12-30

**Authors:** Payel Shee, Tanaya Halder, Chia‐Jung Yang, Nainish Tickoo, Ratiranjan Samal, Ruta Kulkarni, Shishir K. Pandey, Vikas Kashid, Ashis K. Nandy, Arumugam Thamizhavel, Anamitra Mukherjee, Shovon Pal

**Affiliations:** ^1^ School of Physical Sciences National Institute of Science Education and Research An OCC of HBNI Jatni Odisha India; ^2^ Department of Materials ETH Zurich Zurich Switzerland; ^3^ Department of Condensed Matter Physics and Materials Science Tata Institute of Fundamental Research Mumbai India; ^4^ Department of General Sciences (Physics) Birla Institute of Technology and Science, Pilani, Dubai Campus Dubai International Academic City Dubai United Arab Emirates; ^5^ Department of Physics Birla Institute of Technology and Science Pilani, Hyderabad Campus Telangana India; ^6^ Department of Physics Savitribai Phule Pune University Pune India; ^7^ MIE‐SPPU Institute of Higher Education Doha Qatar

**Keywords:** strongly correlated system, rare‐earth intermetallics, ferromagnets, crystal field effect, THz time‐domain spectroscopy

## Abstract

The interplay between magnetic order, charge dynamics, and crystal field excitations underpins the emergent ground states of rare‐earth intermetallics. Using time‐domain terahertz spectroscopy, we probe this coupling in PrSi, a metallic ferromagnet. The optical response exhibits pronounced Drude–Smith behavior over a broad temperature range, indicating persistent carrier scattering. A classical Kondo‐lattice model (CKLM) attributes this non‐Drude conductivity to scattering of itinerant electrons by localized magnetic moments, persisting down to temperatures well below the magnetic ordering scale. At lower temperatures, beyond the scope of CKLM, our experiment reveals that the response is dominated by crystal field excitations, with sharp transitions at 0.6 and 1.54 THz. The mode at 1.54 THz shows a dynamic correlation with the onset of ferromagnetic order, marking the onset of a crystal‐field‐governed low temperature regime.

## Graphical abstract

The interplay between magnetic ordering and crystal electric field (CEF) excitations plays a pivotal role in defining the low‐energy electrodynamics of quantum materials. By probing the temperature‐dependent THz conductivity in a rare‐earth‐based metallic ferromagnet, we uncover a microscopic connection between localized and itinerant electrons, revealing the evolution of electronic localization and the CEF excitation with the onset of magnetic order in the system.

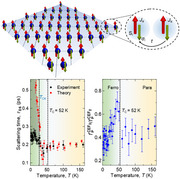

## 1 Introduction

Rare‐earth intermetallic compounds offer a diverse platform to explore strong electronic correlations such as heavy‐fermions [1, 2], topological insulators [3, 4], quantum spin liquids [5, 6] and high‐temperature superconductors [7]. In this class of materials, unpaired electrons in the f‐orbitals of the rare‐earth ions create localized magnetic moments that contribute to the long‐range magnetic order in the material. Here, the direct exchange interaction between the rare‐earth ions is almost negligible because the overlap between the electronic shell of the neighboring ions is usually lower [8, 9]. The magnetic order is thus predominantly mediated by an indirect exchange interaction – the Ruderman–Kittel–Kasuya–Yosida (RKKY) interaction [10, 11, 12]. The ordering temperature (for example, the Curie temperature in ferromagnets) is dictated by the magnitude of the exchange energy scale [13]. The spin‐orbit coupling in addition plays a crucial role in the determination of the Fermi surface alongside the optical and transport properties. The interplay of itinerant carriers with the local moments has a long‐standing history of uncovering phenomena such as Kondo screening and competing magnetic orders [14]. It is well‐known that in turn magnetic order or its lack has a significant bearing on carrier lifetime due to the local‐moment‐mediated scattering and can renormalize electron‐electron interactions [15]. Evidently such correlated systems provide an extended opportunity to explore the interplay between their magnetic properties, carrier scattering rates and the optical properties.

In the context of optical properties, both real ($\sigma_{r}$) and imaginary ($\sigma_{\mathrm{im}}$) parts of the complex‐valued THz conductivity carries information on the materials' electronic response to external light fields [16, 17]. For example, electrons in a homogeneous medium, mostly follow the Drude law where the real part of the THz conductivity in the low frequency regime shows a predominantly higher value [18]. However, for materials where the electrons see an inhomogeneous environment, such as confining structures or localization effects, the conductivity deviates from the regular Drude behavior. In most cases, when the dimension of the confinement becomes comparable to the carrier mean free path, non‐Drude behavior sets in [19]. In such scenarios, the Drude–Smith (DS) model has been a popular choice to account for the experimentally observed non‐Drude‐like THz conductivity in a wide range of materials, such as liquid metals [20, 21, 22], nano‐structured materials [23, 24], disordered crystals [25, 26, 27] and molecular networks [28, 29, 30]. The DS model is in fact an extension of the regular Drude model with the introduction of a “localization parameter” that quantifies the fraction of original velocity retained by the electrons after each scattering, or equivalently, the degree of backscattering in the system. In magnetic materials, Coulomb‐interaction‐mediated electronic scatterings and the exchange‐interaction‐driven magnetism are strongly correlated to each other. In systems with large magnetic moments allowing these to be treated classically, the relative orientation of the spins, in fact, influences the conductivity of a ferromagnetic metal. Above the Curie temperature, when the spins are randomly oriented, more scattering is introduced in the system, causing the system's conductivity to deviate from the ideal Drude behavior. Below Curie temperature the spins align, which should ideally reduce the scattering rate. In reality, however, the emergence of magnetic domains could affect the overall transport as well as the optical properties. It is known that the exchange interactions between the itinerant and the localized electrons often give rise to anomalous conductivity in ferromagnetic materials [31, 32, 33]. In addition, the presence of strong spin‐orbit coupling can also lead to non‐Drude transport, which has been demonstrated in ferromagnetic iron [34].

While on one hand the magnetic ordering interferes with the Coulomb interaction in governing the transport characteristics of magnetic systems, the crystal electric field (CEF) on the other hand can potentially influence the magnetic ground state and anisotropy in these materials that concomitantly modifies the optical conductivity. Due to the periodic arrangement of the ligands/ions in the crystal structure, the CEF arises in the system, which breaks the degeneracy of f‐orbitals in a rare‐earth system. Depending on the total angular momentum (J) of the rare‐earth ion, the CEF splits the ground state into (2J+1) sub‐levels, where the spacing between the levels lies in the meV range. By breaking the degeneracy of the ground state, the CEF partially quenches the orbital contribution of the magnetic moment, resulting in a suppression of the total magnetic moment at temperatures close to the energy scales of the CEF splittings [35]. The CEF thus becomes a pivotal factor in deciding the magnetic ground state of the rare‐earth based systems. The interplay between the RKKY interaction and the CEF energy scales gives rise to several fascinating phenomena, namely, the exotic magnetic behavior in rare‐earth based triangular‐lattice quantum spin liquid systems [36, 37], spin reorientation phase transition in rare‐earth orthoferrites [38] and the renormalized energy‐scales of the CEF satellite states at quantum critical point in rare‐earth based Kondo systems [39, 40, 41]. There are, however, limited work on the study of how the underlying CEF environment interferes the magnetic ordering in a rare‐earth intermetallic compound having a ferromagnetic ground state.

**FIGURE 1:**
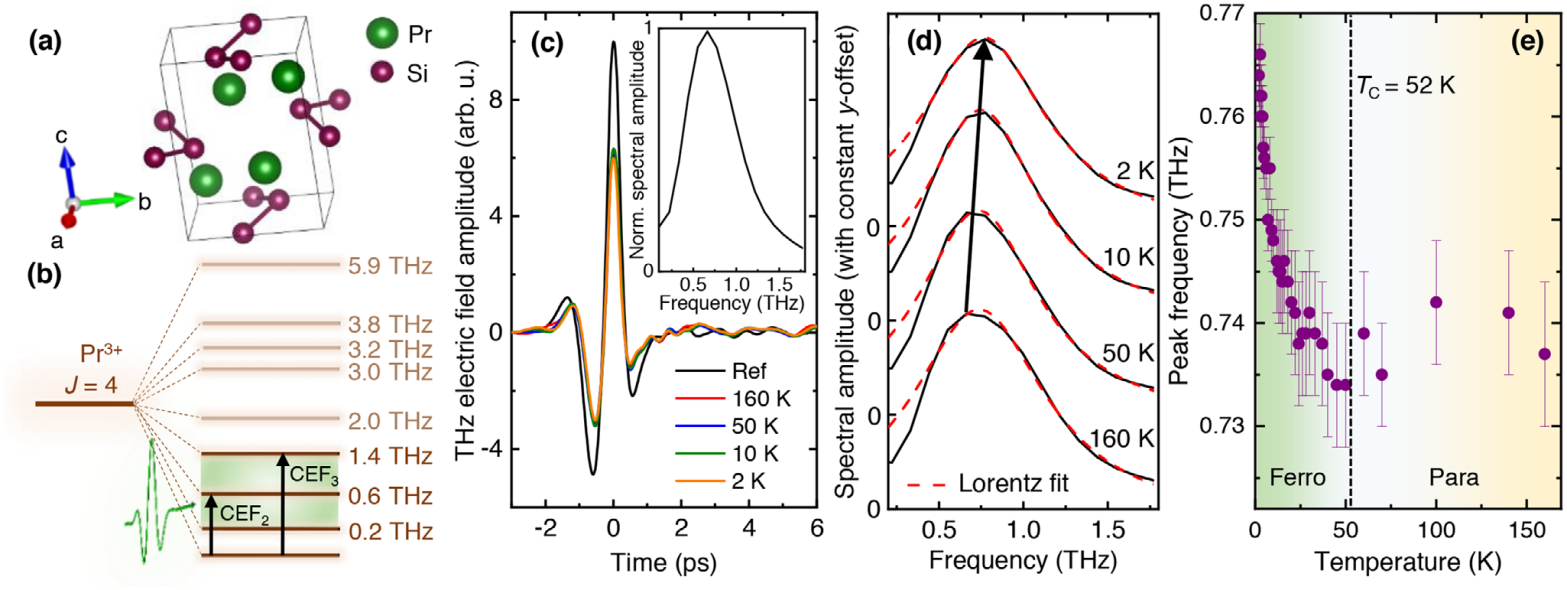
(a) A schematic of the orthorhombic crystal structure of PrSi. (b) A schematic showing the splitting of the J=4 level of the central ion ${\mathrm{Pr}}^{3+}$ into 2J+1=9 sub‐levels of distinct energy values, all in the THz range. The incident THz pulse interacts with the lowest four CEF multiplets. (c) THz transients reflected from the reference Pt‐mirror and the PrSi sample at different temperatures. **(Inset)** The incident THz spectra. (d) The spectral amplitude corresponding to the sample at the corresponding temperatures shown in (c), obtained from the Fourier transform of the time transients. The zero‐level corresponding to each spectra is marked. The red‐dashed lines refer to the Lorentzian fitting of the respective spectra. The arrow indicates the peak shift as we lower the temperature. (e) The temperature‐dependent peak frequency corresponding to (d), which shows a blue shift as we enter in the ferromagnetic phase of the material. Here, the error bars are the standard errors from the modeling of the spectra. The vertical dashed line indicates the Curie temperature, $T_{C}=52$ K.

In this article, we present the influence of magnetic ordering on the THz conductivity in a rare‐earth based metallic ferromagnet exhibiting dominant crystal field environment at lower temperatures. For our investigations, we chose a Pr‐based intermetallic compound, PrSi, which is metallic at all temperatures. PrSi crystallizes in an orthorhombic FeB‐type crystal structure, as shown in Figure 1a, with the space group Pnma. Here, Pr is a rare‐earth element, having a partially‐filled 4f orbital, which creates the magnetic moment in the material. Due to the Russel‐Saunders coupling scheme, the central ion ${\mathrm{Pr}}^{3+}$ gets a total angular momentum of J=4, i.e, this ion posses a (2J+1)=9‐fold degeneracy in its ground state. In presence of CEF, the ground state degeneracy splits into 9 singlets with an overall spacing of 284 K [42]. The ground and the first excited states are, however, separated by only 9 K which is essentially responsible for the magnetic ordering in the material [43]. This material undergoes a paramagnetic to ferromagnetic phase transition below $T_{C}=52$ K [44, 45]. From earlier reports, we found that the [010]‐axis is the easy magnetization axis of the material. The magnetic behavior in the other two axes are relatively complex. In particular, below 10 K, the in‐plane magnetization shows an anomalous behavior due to the presence of the CEF transition in the system. Through theoretical modeling of the magnetic susceptibility data, the CEF transitions in PrSi are predicted to lie in the THz range, specifically in the range 0.18−5.9 THz [42], see Figure 1b. Using THz time‐domain spectroscopy, we observed an overall predominance of non‐Drude behavior in the THz conductivity on either side of the phase transition. The non‐Drude behavior supports the existence of carrier backscatterings in the system on either side of the phase transition. This behavior is phenomenologically explained using the classical Kondo lattice model (CKLM) up to a temperature of $T_{\mathrm{CK}}$ well below the $T_{C}$ of the material. Since the phenomenological CKLM is used to model the magnetic scattering effects, it misses out on the CEF effects. In the experiment we continue to observe the non‐Drude behavior till the lowest temperature measured. We argue that for temperatures lower than $T_{\mathrm{CK}}$, the carrier scattering introduced by the CEF environment most likely takes over leading to the observed non‐Drude behavior. Upon a careful scrutiny of the conductivity spectra, we find evidences of CEF states that was theoretically predicted earlier [42]. From the spectral weight analysis, we find that the relative occupation of the third CEF state corroborates the $T_{C}$ of the material. Our observations thus highlight the underlying coupling between the CEF environment and the magnetic ordering in the material.

## 2 Results and Discussion

Temperature‐dependent THz reflection measurements were performed to investigate the nature of the low‐energy optical conductivity as PrSi goes through the paramagnetic to ferromagnetic phase transition. The exemplary THz time transients reflected from the PrSi single‐crystal for a few temperatures along with the reference Pt‐mirror are shown in Figure 1c. The inset in Figure 1c shows the typical THz spectrum that is incident on the sample. In addition to an overall absorption, we can observe the subtle indications of additional oscillatory features in the tail of the reflected THz pulses. The spectral response at different temperatures is obtained by taking a fast Fourier transform of the time transients, some of which at few distinct temperature points are shown in Figure 1d. We find that the spectral peak shows a continuous blue shift as the sample enters the ferromagnetic phase, i.e., below $T_{C}=52$ K as shown in Figure 1e. We quantify this shift to be around 30 GHz by using a Lorentzian function to fit the temperature‐dependent reflection spectra. We speculate that the blue shift is associated with the enhanced RKKY‐mediated exchange energy as material enters deeper into the ferromagnetic phase. Such exchange‐mediated spectral blue shifts have been reported earlier in metallic [39, 41] and semiconductor [46] systems with antiferromagnetic ground state or in Mott systems [47].

**FIGURE 2:**
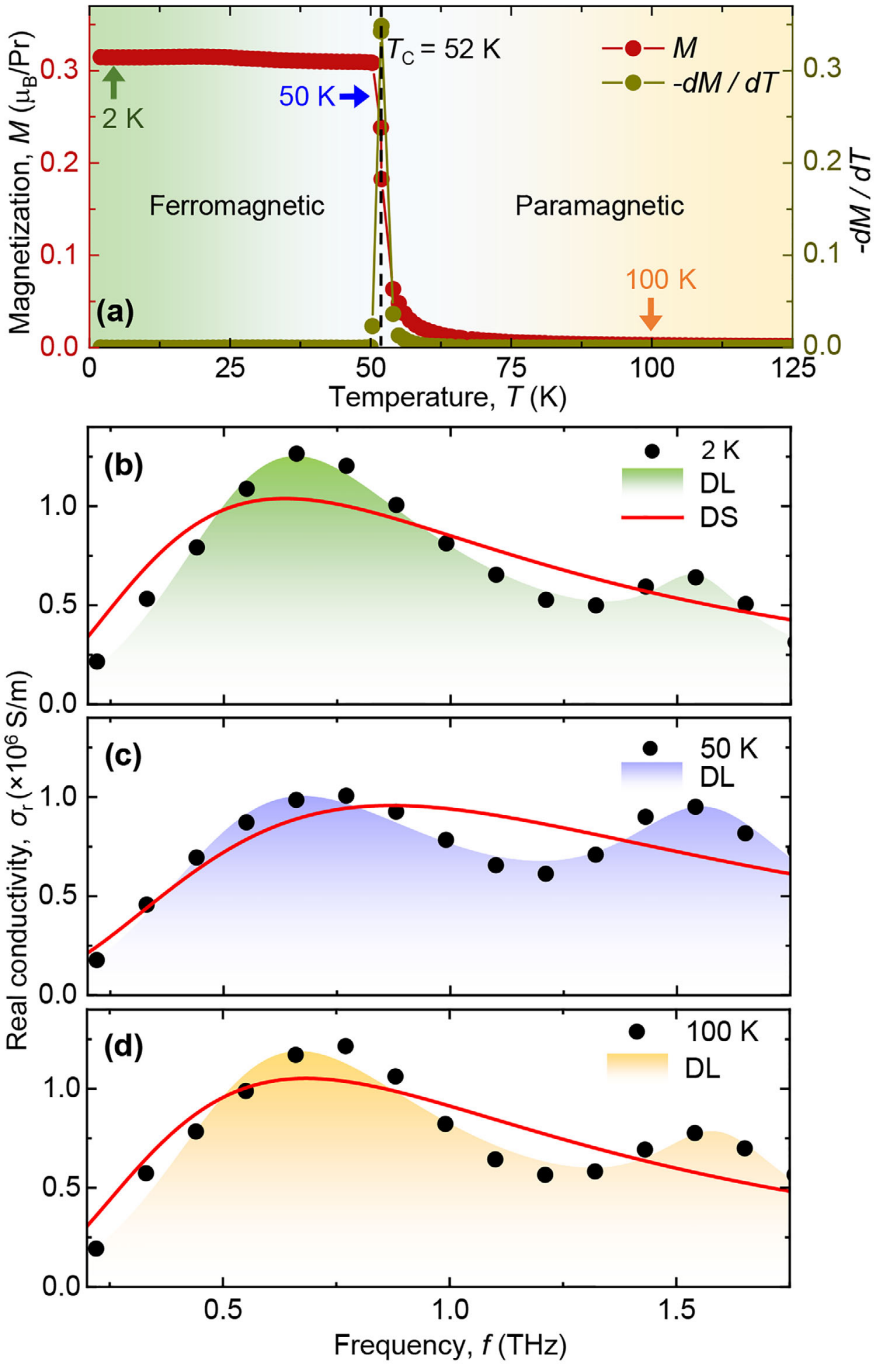
(a) Temperature‐dependent magnetization and its derivative, showing the $T_{C}$ of the material at 52 K. The real part of the THz conductivity as a function of frequency for (b) 2 K, (c) 50 K, and (d) 100 K, respectively. The solid‐red lines represent the Drude–Smith (DS) model. The colored areas represent the fitting using the double‐Lorentz (DL) oscillator model.

The $T_{C}$ of 52 K [42, 43] was verified for the current sample by performing the magnetization measurements using a Quantum Design SQUID magnetometer. Figure 2a, shows the temperature‐dependent magnetization along the easy axis (i.e., [010] direction) in presence of an external out‐of‐plane magnetic field of 0.05 T. The magnetization shows a sharp increase as the system evolves from paramagnetic phase (yellow shaded region) to the ferromagnetic phase (green shaded region). The Curie temperature quantified by taking a temperature derivative of the magnetization (i.e., dM/dT) as shown in Figure 2a, which shows a peak at 52 K, marking the Curie temperature for our single‐crystal PrSi. To understand the impact of magnetic ordering on the electron‐electron interaction from the temperature‐dependent reflected THz responses, we extract the real ($\sigma_{r}$) and imaginary ($\sigma_{\mathrm{im}}$) parts of the THz conductivity. This is obtained using Fresnel's equation for the p‐polarized light, as per the geometry of our experiments. Further elaborate details on the analysis procedure can be found elsewhere [41].

PrSi by nature is metallic across the phase transition, indicating that one would expect the conductivity to follow Drude‐like behavior. This is, however, in striking contrast to what we have observed in our experiments. We find that the conductivity is predominantly non‐Drude‐like. Deviations from the Drude behavior have been previously reported in several other metallic systems, such as heavy‐fermions [41], high‐$T_{c}$ superconductors [48] and magnetic metals [33]. Figure 2b–d show, respectively, the real part of the THz conductivity at 2, 50, and 100 K, corresponding to three different points in the PrSi phase diagram, namely, the ferromagnetic side, close to the transition point and the paramagnetic side. We use two different approaches to model our observation. In the first approach, we use the Drude–Smith (DS) model (as indicated by the red‐solid lines in Figure 2b–d), motivated from the fact that the system hosts strong electronic interactions between the localized 4f‐electrons of Pr and the itinerant conduction electrons. In the second approach, we use a double‐Lorentz (DL) model (shown as colored areas in Figure 2b–d) to precisely account for the observed conductivity peaks, i.e., at around 0.6 and 1.54 THz. The possibility of magnons in this frequency range can be safely ignored as discussed in Ref. [42]. We have also verified the absence of phonons in the measured frequency range using density‐functional theory calculations (see the phonon calculations in Section S1 of the Supporting Information). We, thus, associate the observed peaks to the CEF states that has been theoretically proposed earlier [42] to lie in the same frequency range.

We first scrutinize the real part of the THz conductivity using the DS model, where the complex conductivity, ${\tilde{\sigma }}_{\mathrm{DS}}\left(\omega \right)=\sigma_{r}\left(\omega \right)+i\sigma_{\mathrm{im}}\left(\omega \right)$ is given by,

(1) $$\begin{array}{c} {\tilde{\sigma }}_{\mathrm{DS}}\left(\omega \right)=\frac{\omega_{p,\mathrm{DS}}^{2}\varepsilon_{0}\tau_{\mathrm{DS}}}{1-i\omega \tau_{\mathrm{DS}}}\left[1+\frac{c}{1-i\omega \tau_{\mathrm{DS}}}\right]. \end{array}$$

**FIGURE 3:**
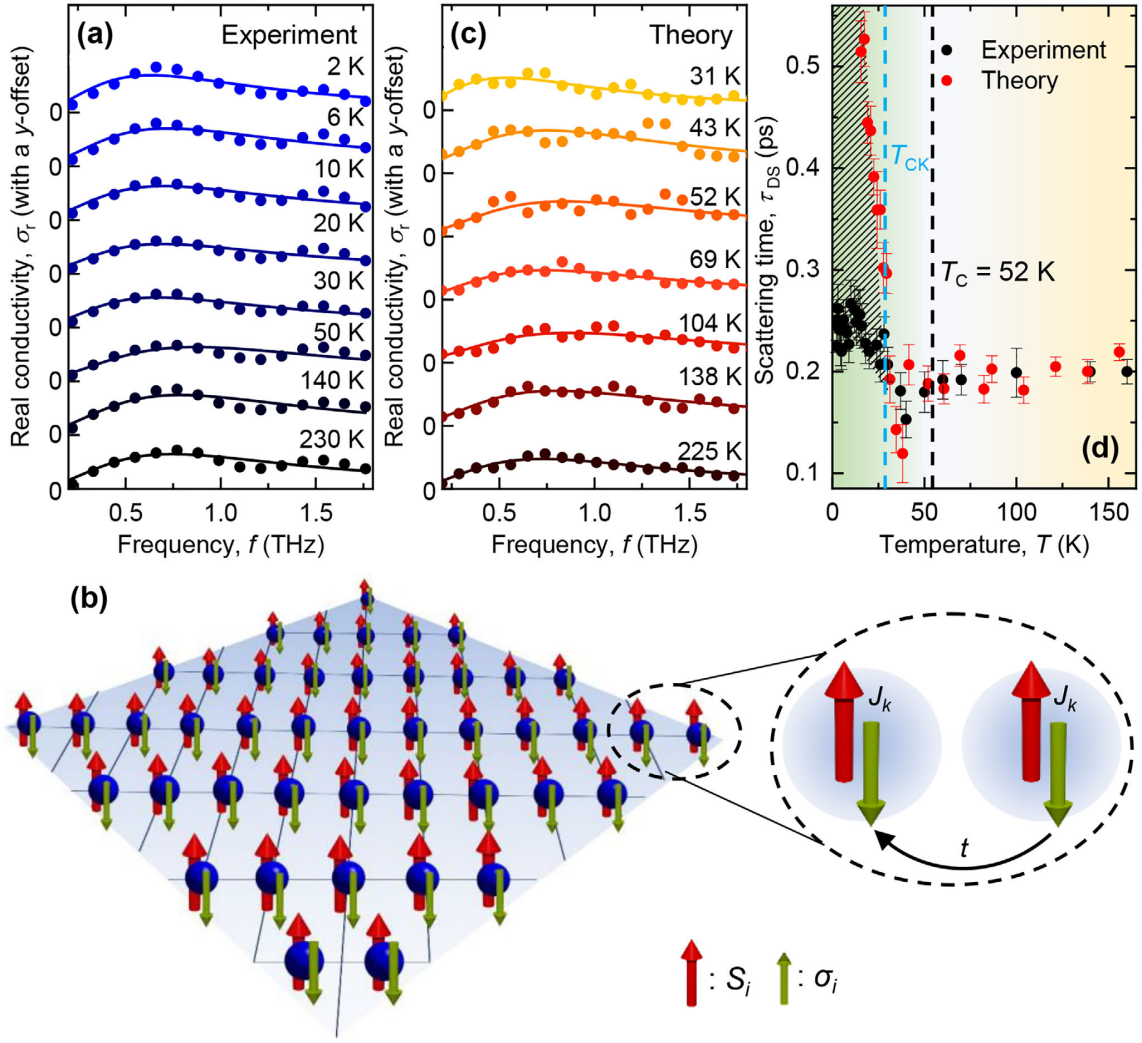
(a) Temperature‐dependent real part of the THz conductivity obtained from our experiments. The solid lines represent the fitting using Drude‐Smith (DS) model. (b) A schematic representation of the CKLM, where the localized 4f‐moments (red arrows, $S_{i}$) are coupled to the itinerant electrons (green arrows, $\sigma_{i}$) at every lattice site. **(Inset)** The localized and itinerant electrons are coupled with a coupling strength $J_{k}$ and t being the nearest‐neighbor hopping parameter. (c) Temperature‐dependent real part of the THz conductivity obtained from our theoretical CKLM. The solid lines represents fitting using Drude–Smith model. (d) The scattering time $\tau_{\mathrm{DS}}$ obtained by fitting the Drude–Smith models corresponding to the experimental and theoretical conductivity spectra. The yellow and green‐shaded regions show the paramagnetic and the ferromagnetic phases, respectively. The vertical black‐dashed line marks the $T_{C}$ of the material. The vertical cyan‐dashed line marks the temperature below which the conductivity obtained from CKLM deviates from the experimental observations. Here, the error bars represent the standard errors from the DS‐modeling of the experimental and theoretical conductivity data.

Here, $\omega_{p,\mathrm{DS}}$ (= 2$\pi f_{p,\mathrm{DS}}$) denotes the plasma frequency, $\tau_{\mathrm{DS}}$ is the scattering time, $\varepsilon_{0}$ is the vacuum permittivity, and c is the localization parameter, ranging from −1 to 0. A value of c=−1 corresponds to the maximum backscattering, where the electron reverses its direction completely after a scattering event. On the other hand, with c=0 the DS model (i.e., Equation 1) reduces to the standard Drude model, with zero backscattering events. Figure 3a shows the real part of the THz conductivity at several temperatures along with the DS model as solid‐lines. It is quite evident the DS model remains dominant throughout the entire temperature range. Upon fitting the real part of Equation 1 to the experimental data, we extracted the temperature‐dependent scattering time ($\tau_{\mathrm{DS}}$) that carries the information on the nature of electronic interactions in the system, see the black dots in Figure 3d. We find that above $T_{C}$ (i.e., in the paramagnetic phase), the scattering time is almost temperature‐independent and is close to ≈0.2 ps. Below $T_{C}$, however, $\tau_{\mathrm{DS}}$ increases and settles at a slightly higher value (≈0.25 ps). Higher scattering time (alternately, reduced scattering rate) in the ferromagnetic phase clearly signals the existence of an intricate relationship between the electronic interactions and the magnetic ordering in the system. Such correlation (namely, exchange‐induced electronic scattering) often leads to anomalies in DC resistivity [49, 50]. It is striking to note that the $\tau_{\mathrm{DS}}$ corroborates the $T_{C}$ of the material to a very good extent. The temperature‐dependent imaginary parts of the THz conductivity and the corresponding results obtained from the DS model fitting of the imaginary part of Equation 1 are shown in Section S2 of Supporting Information. We note that we obtained identical values of scattering times from the fitting of the imaginary THz conductivity. We have, further, traced the temperature evolution of the localization parameter, c, obtained from the Drude‐Smith model. This parameter gives a qualitative idea on the localization effects and hence a measure of the deviation from the ideal Drude transport. We find that the change in the localization parameter is maximum close to the phase transition, shown in Figure S3 of the Supporting Information. This would indicate that close to the phase transition temperature, the material goes through a dramatic change in the electronic environment and an onset of the formation of confining structures such as domains, which potentially acts as source for an overall increase in the material's entropy and thereby sourcing the magnetocaloric effect – a phenomenon that has been reported to occur in the same temperature range [42].

To gain a qualitative understanding on the correlation between the magnetic ordering and the electronic scattering rates, we formulated a classical Kondo lattice model (CKLM) at the phenomenological level. Our model considers the localized moments to be periodically arranged on a 2D lattice, which are further coupled to the itinerant electrons. This is schematically shown in Figure 3b. In the rare earth materials, such local moments arise due to the Hund's coupling in the f‐shells. Within the CKLM, the Hamiltonian H is given by

(2) $$H=-\sum_{<i,j>,\sigma }\left(t_{ij}c_{i\sigma }^{†}c_{j\sigma }+h.c\right)+J_{k}\sum_{i}S_{i}\cdot \vec{\sigma_{i}}-\mu \sum_{i}n_{i}.$$

The first term of the Hamiltonian corresponds to the nearest‐neighbor hopping that describes the motion of the conduction electrons in the lattice. The second term describes the coupling between the localized 4f‐spins and the itinerant conduction electrons in the system. The last term is used to control the conduction electron density $n_{i}$, given by $n_{i}=\sum_{\sigma }c_{i\sigma }^{†}c_{i\sigma }$ through the chemical potential μ. Here, $t_{ij}=t=1$ is a uniform hopping amplitude, which sets the energy scale of the problem, $c_{i\sigma }$ and $c_{i\sigma }^{†}$ are the fermion annihilation and creation operators, respectively, and $J_{k}$ is the local electron‐spin coupling strength. In our model, we consider the local moments $S_{i}$ to be classical (mimicking the Pr f‐orbital moments), while $\vec{\sigma_{i}}$ is the spin of the conduction electron at site i, given by $\vec{\sigma_{i}}=\sum_{\alpha ,\beta }c_{i\alpha }^{†}\frac{\sigma_{\alpha \beta }}{2}c_{i\beta }$, where the σ is composed of Pauli matrices. The model possesses full SU(2) symmetry and has continuous O(3) symmetry. It is well‐known that such a model cannot have long‐range magnetic order at finite temperatures. We employ an infinitesimal symmetry breaking magnetic field to stabilize magnetic order. We have checked the robustness of our results by varying the symmetry‐breaking field strength. Using a reasonable value of t=0.07eV, the value of scattering time, $\tau_{\mathrm{DS}}$ is matched with the experiments at higher temperatures. All temperature and frequency scales in the model are converted to the respective experimental units using this value of t. We note that the results reported here hold for $J_{k}/t∼1.3$ to 1.5 on a square lattice where the ground state in the quantum limit is in the RKKY regime [51, 52] and does not predict Kondo screening, consistent with earlier reports on PrSi [42].

Our model that underpins the coupling between the classical‐spins and the itinerant fermions falls in the category of spin‐fermion models [53, 54, 55], which is known [56, 57] to be solved using classical Monte–Carlo for annealing the classical degrees of freedom coupled with exact diagonalization for the fermion problem, allowing access to large lattice sizes [58, 59]. Using this exact diagonalization+Monte‐Carlo (ED+MC) approach (as described in the methods section), we evaluate the frequency‐resolved complex THz conductivity at several temperatures across the phase transition, the real part of which are shown in Figure 3c. We find that above $T=T_{\mathrm{CK}}$, the CKLM provides a very good agreement with our experimental observations, as shown by the red dots in Figure 3d. This clearly indicates that the low‐energy transport is governed by the scattering between the localized 4f spins and the itinerant electrons in our material system that ultimately results in the non‐Drude nature of the transport. In the paramagnetic phase, when all the spins are randomly oriented, the electronic scattering within the system is high, i.e., the time between two scattering events (scattering time) is less. Below the Curie temperature, however, when the spins start to align, the electronic scattering decreases, leading to an increase of the scattering times. We note that below $T_{\mathrm{CK}}$ the scattering time obtained from the CKLM model deviates from the experimental ones. This is because in the CKLM, the scattering time diverges [31] once the classical spins order into a long‐range uniform ferromagnet (for $T<T_{\mathrm{CK}}$). The model also allows us to calculate the inverse participation ratio (IPR) to qualitatively explore the localization properties of the itinerant electrons across the phase transition, see Section S4 of the Supporting Information for further details. We found that as we approach the $T_{C}$ from the magnetic side, the enhanced disordering of the core spins increases the scattering, leading to an increased value of the IPR, evidently signifying an increased carrier localization, concomitant with the systematic suppression of the Drude weight as ω→0 [60] for $T>T_{C}$.

Our experiments however continues to exhibit non‐Drude behavior. It is natural to expect the persistence of magnetic domains in the ferromagnetic phase in our experiments down to much lower temperature than in the CKLM, the dimensions of which lie in the same order of magnitude as the carrier mean free path [61]. We anticipate that this could also potentially introduce scattering centers in the magnetic phase at lower temperatures. Further, as discussed below, our experiments suggest another source of non‐Drude behavior originating from the underlying crystal field environment, which is, however, beyond the scope of the simple CKLM.

**FIGURE 4:**
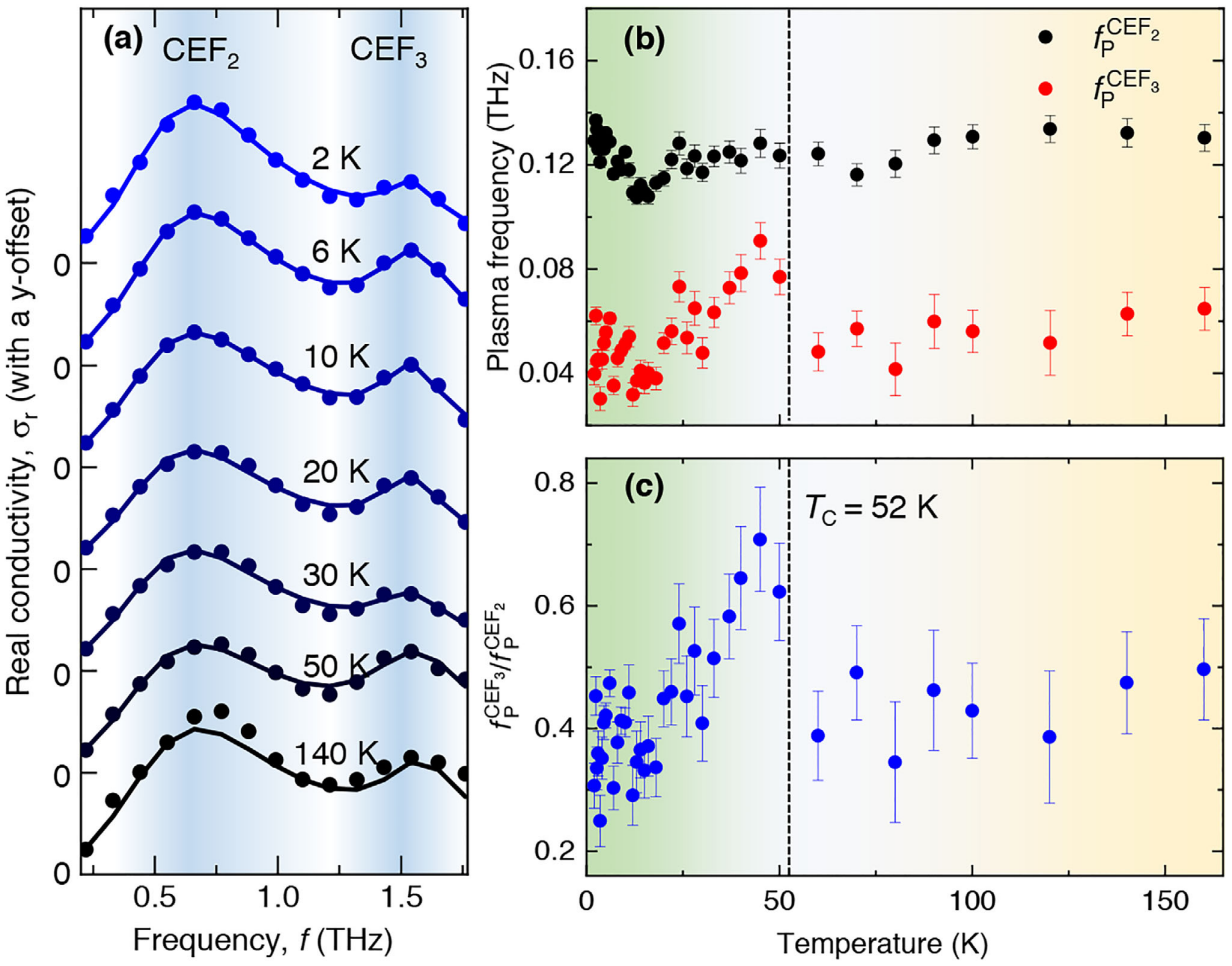
(a) Real part of the THz conductivity at different temperatures. The solid lines represents fitting using the double‐Lorentz (DL) model. (b) The plasma frequency corresponding to both oscillators, plotted as a function of temperature. (c) The ratio of the plasma frequencies in (b). The yellow‐ and green‐shaded regions show the paramagnetic and the ferromagnetic phase, respectively, while the vertical dashed‐line marks the Curie temperature of the system. Here, the error bars are the standard errors from the modeling of the experimental conductivity data.

To substantiate the presence of crystal field environment, we choose a different approach where we take a closer look at the temperature‐dependent spectral features in the real part of the THz conductivity. We find that the conductivity peaks at two frequencies, namely 0.6 and 1.54 THz (see Figure 4a). The peak positions are in good agreement with the reported second and third CEF transitions in PrSi [42], see Figure 1b. Since the first CEF state lies at 0.2 THz, it is beyond our spectral resolution to probe it. We, thus, capture the next two CEF transitions at 0.6 and 1.54 THz. For our reference in the subsequent discussions, we name these transitions as ${\mathrm{CEF}}_{2}$ and ${\mathrm{CEF}}_{3}$. From the experimental real conductivity, shown in Figure 4a, we observe that the spectral amplitude of both these peaks display a distinct temperature dependence. We notice that the spectral amplitudes of the two peaks become almost comparable near $T_{C}$. To quantify this behavior, we fit the experimental curves using double‐Lorentz (DL) oscillator model, shown by the solid lines in Figure 3a that is given by,

(3) $$\sigma_{\mathrm{DL}}\left(\omega \right)=\sum_{i=1}^{2}\varepsilon_{0}\omega_{pi,\mathrm{DL}}^{2}\frac{\omega \tau_{i,\mathrm{DL}}}{\omega +i\tau_{i,\mathrm{DL}}\left(\omega_{0i}^{2}-\omega^{2}\right)}.$$

Here, $\omega_{pi,\mathrm{DL}}=2\pi f_{pi,\mathrm{DL}}=\sqrt{n_{i}e^{2}/m^{*}\varepsilon_{0}}$ is the plasma frequency. $\tau_{i,\mathrm{DL}}$ is the relaxation time, and $\omega_{0i}$ is peak frequency corresponding to the DL model (where i=1,2). $n_{i}$ is the electronic density involved in the CEF transition, e is the electronic charge, and $m^{*}$ is the effective mass of electrons. From our model fitting, we extracted the plasma frequency, which is a measure of the density of bound electrons per unit volume that contribute to the specific resonant CEF state absorption. The respective plasma frequencies are plotted in Figure 4b as a function of temperature. We find that the plasma frequency corresponding to the ${\mathrm{CEF}}_{2}$ is always higher than the ${\mathrm{CEF}}_{3}$, which implies a higher density of electrons involved in ${\mathrm{CEF}}_{2}$ transition as compared to the ${\mathrm{CEF}}_{3}$ transition. This is evident since ${\mathrm{CEF}}_{2}$ is energetically lower than ${\mathrm{CEF}}_{3}$. Surprisingly, close to the phase transition, the density of electrons involved in ${\mathrm{CEF}}_{3}$ transition increases significantly, as plotted in Figure 4c. We have alternately verified our observations by calculating the frequency‐integrated spectral weights using the Kubo's formula (see Section S6 of Supporting Information). On lowering the temperature below $T_{C}$, the ${\mathrm{CEF}}_{3}$ occupation relaxes back. Our results clearly highlights an intrinsic correlation between the CEF occupation and the onset of magnetic ordering. We note that while the CKLM works primarily for the high temperature phase, the Lorentz oscillator model works throughout the entire temperature range. The line‐widths (i.e., $1/\tau_{i,\mathrm{DL}}$) of both the CEF peaks further substantiates the model validation, where we can clearly note an increase in its value with the onset of the magnetic order in the system, see Section S7 of Supporting Information. These low temperature observations also highlight the need of incorporating CEF in a Kondo lattice model treated with more sophisticated techniques such as Abrikosov fermion approach [62] to retain low temperature quantum fluctuations, providing an ample scope for further work along this direction.

As PrSi goes from the paramagnetic (disordered) to the ferromagnetic (ordered) state, there is a change in the entropy associated with it, which leads to a change in the temperature of the material. The crystal electric field plays an important role in this process because it induces strong magnetic anisotropy in the system [63, 64, 65]. The presence of the strong interplay between the crystal field and the magnetic ordering, as highlighted in our work through an enhanced occupation of third CEF state near $T_{C}$ (shown in Figure 4c), strongly corroborates the previous observation of giant magnetocaloric effect in these materials [42, 43], an analogy of which is drawn in Figure S6b of the Supporting Information. It is known that for rare‐earth materials to have a high magnetocaloric property, the rare‐earth should possess a high angular momentum with a high exchange interaction energy [66], both of which are reported in PrSi [42].

## 3 Conclusion

In conclusion, our investigation on the temperature‐dependent THz conductivity in a metallic ferromagnet, PrSi, provides a fresh outlook on the intrinsic interplay of crystal field environment on the magnetic ordering and the correlation between the magnetic $T_{C}$ and the electronic scatterings. In particular, we observed a predominant non‐Drude transport across the paramagnetic to ferromagnetic phase transition. This non‐Drude response is associated with the carrier scattering events within the system, where the carrier scattering rate increases below $T_{C}$. The behavior is phenomenologically modeled to a very good extent using a classical Kondo‐lattice model, which highlights scattering interactions between the localized 4f‐spins of ${\mathrm{Pr}}^{3+}$ and the itinerant electrons. The model reproduces the experiments until a temperature that we mark as $T_{\mathrm{CK}}$, below which we argue that the underlying crystal field environment takes over. A more elaborate analysis of our conductivity data shows the presence of two CEF peaks that reveals a distinct temperature behavior, where the occupation of third CEF state peaks close to the phase transition. Our experiments clearly signals the presence of a dynamic correlation between the magnetic ordering and the crystal field environment. This correlation is, however, not unique to PrSi and can be extended to other rare‐earth silicides RSi (R= Nd, Ho, Dy, and Tb) [67, 68, 69, 70, 71], ${\mathrm{Pr}}_{5}\mathrm{Si}$ 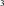 [72, 73], RNi (R= Pr, Gd, Tb, Dy, Ho, and Er) compounds [13, 74, 75, 76] and RAl 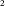 compounds [63]. Given the wide applicability, we anticipate that our results open up an ample scope for both theoretical and experimental developments on identifying such intrinsic correlations between the spin and charge degrees of freedom in a broader class of strongly interacting systems.

## 4 Experimental and Numerical Methods

**Sample Preparation** Single crystal of PrSi was synthesized using the Czochralski pulling technique in a tetra‐arc furnace (Technosearch Corporation, Japan), maintained under an argon atmosphere. Since PrSi melts congruently at 1657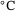, the high‐purity starting elements, Pr (99.9%) and Si (99.999%) were taken in a stoichiometric 1:1 ratio. The mixture was melted multiple times in the tetra‐arc furnace to ensure compositional uniformity. A tungsten rod was used as a seed crystal, which was pulled at a constant rate of 10 mm/h. Samples from the same ingot were previously used for the investigation of electrical resistivity, magnetization, specific heat, and magneto‐caloric effects [42].

For our current investigations, we cut the sample surface oriented perpendicular to the crystallographic b‐axis using a wire electric discharge machine. The sample surface is polished using colloidal silica, ensuring that any residual roughness is within the sub‐micron range (i.e., $<<\lambda_{\mathrm{THz}}$). Given the metallic nature of PrSi across all temperatures, the experiments are carried out in the reflection geometry using linearly‐polarized THz radiation with a spectral range of 0.2−2.5 THz, incident at an angle of 45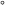. The electric field vector of the incident radiation lies within the crystallographic ac‐plane. The sample is then mounted in a temperature‐controlled helium reservoir cryostat for the temperature‐dependent studies.

**THz Time‐Domain Experiments** Single‐cycle THz pulses are generated by optical rectification using a 0.5 mm thick ZnTe crystal oriented along the (110)‐plane, utilizing 90% of the output from a Ti:Sapphire laser (central wavelength 800 nm, pulse duration 120 fs, repetition rate 1 kHz, and a pulse energy 2 mJ). The remaining 10% of the beam is used as the gating pulse for free‐space electro‐optic detection of the reflected THz pulses. Both the reflected THz pulse and the gating beam are allowed to co‐propagate through a second (110)‐oriented ZnTe detection crystal. The THz‐induced ellipticity in the gating beam is analyzed using a combination of a quarter‐wave plate, a Wollaston prism, and a balanced photodiode detector. To suppress the Fabry‐Perot resonances caused by the internal reflections within the 0.5‐mm thick detection crystal, a 2‐mm thick THz‐inactive (100)‐oriented ZnTe crystal is optically bonded to its rear surface, thereby extending the accessible time window. All measurements are performed in an inert nitrogen environment. A 15‐nm Pt film grown on a quartz substrate is used as the reference and placed next to the sample within the cryostat to ensure identical experimental conditions.

**Monte Carlo Method** The classical Kondo lattice model involves both classical and quantum degrees of freedom that are coupled to each other. For this purpose, we use the ED+MC method to study the model at finite temperatures [77, 78]. Our method follows a two‐step process: (i) update the classical spin at a site, (ii) exactly diagonalize the full Hamiltonian for fixed classical background. We start by generating a random configuration of the spins on the entire lattice, say $\left\{S_{i}\right\}$. The $S_{i}$ has a fixed value and it can orient in any arbitrary direction in the 3‐D space. The Hamiltonian is then generated for this classical configuration, $H(\left\{S_{i}\right\})$, and is diagonalized to find the energy, say $E_{1}$. We then propose an update at site, say j, and update the spin configuration on that particular site $S_{j}$ and re‐diagonalize the Hamiltonian with that updated spin configuration to get an energy $E_{2}$. If the new energy with updated spin configuration is less than the old energy, $(E_{1}-E_{2})>0$, then the update is accepted and the total spin configuration, $\left\{S_{i}\right\}$, is updated. If $(E_{1}-E_{2})<0$, then the update is accepted or rejected based on the Boltzmann probability. At any particular temperature this process is repeated by sequentially visiting all the lattice sites. This constitutes a single system sweep. A thermalized regime is reached after a large number of system sweeps at a fixed temperature as discussed below. Once we reach the thermalized regime, we calculate the desired observables from the equilibrium configurations.

In our calculations, we start the simulation at a high temperature with a random spin configuration and then cool down the system in small steps of temperature so that the system does not get stuck in an intermediate metastable state. Here, the simulations are performed on an 8×8 square lattice, using 2000+2000 Monte–Carlo steps, where the first 2000 steps are used to thermalize the system and the later 2000 steps are used to calculate the observables. We calculate the observables after every 10^th^ step in the later part to avoid any autocorrelation.

For the characterization of the magnetic ordering in our system, we look at the magnetic structure factor S(q), given by

$$S\left(q\right)=\frac{1}{N^{2}}\sum_{i,j}e^{iq\cdot (r_{i}-r_{j})}\langle S_{i}\cdot S_{j}\rangle ,$$

where q=0,0 is the wave‐vector considered, as we are interested in the ferromagnetic order. N is the dimension of the system. $S_{i}$ and $S_{j}$ are the spins at the i−th and j−th site, respectively. $r_{i}$ and $r_{j}$ give the position of the respective spins. The structure factor gives an estimation for the ordering temperature, see Figure S2 of the Supporting Information. We further evaluate the optical conductivity of the system, using

$$\sigma \left(\omega \right)=\frac{\pi e^{2}}{N\hbar a_{0}}\sum_{\alpha \beta }\left(n_{\alpha }-n_{\beta }\right)\frac{|f_{\alpha \beta }{|}^{2}}{\varepsilon_{\beta }-\varepsilon_{\alpha }}\delta \left(\omega -\left(\varepsilon_{\beta }-\varepsilon_{\alpha }\right)\right),$$

where $f_{\alpha \beta }$ are the matrix elements of the current operator, explicitly given by $f_{\alpha \beta }=\langle \psi_{\alpha }|J_{x}|\psi_{\beta }\rangle$. $J_{x}$ being the current operator, given by

$$J_{x}=-ia_{0}t\sum_{i,\sigma }\left(c_{i,\sigma }^{†}c_{i+a_{0}\hat{x}}-h.c.\right).$$

Here, $|\psi_{\alpha }\rangle$ and $\varepsilon_{\alpha }$ are the single‐particle eigenstates and eigenvalues, respectively. $n_{\alpha }=f\left(\mu -\varepsilon_{\alpha }\right)$ is the Fermi function and $a_{0}$ is the lattice parameter.

## Author Contributions

All authors contributed to the discussion and interpretation of the experiment and to the completion of the manuscript. P.S. and C.J.Y. performed the experiments. P.S. and N.T. performed the data analysis. R.K. and A.T. provided the samples. R.S. performed the magnetization measurements. T.H., V.K., A.K.N. and A.M. developed the theoretical model, while T.H. and P.S. analyzed the theoretical results. S.K.P. and A.K.N. performed the phonon calculations. A.T. and S.P. conceived the project while S.P. supervised the experiments. P.S., T.H., A.M. and S.P. drafted the manuscript.

## Conflicts of Interest

The authors declare no conflict of interest.

## Supporting information

Supporting Information

## Data Availability

The data that support the findings of this study are available from the corresponding author upon reasonable request.
