# Supplementary material for: The Interplay of Magnetic Order with the Electronic Scattering and Crystal‐Field Effects in a Metallic Ferromagnet
Source: Adv Sci (Weinh). 2025 Dec 30;13(13):e17704. doi: 10.1002/advs.202517704 (PMC12955905; doi:10.1002/advs.202517704)
Supplement: Supplementary file 1 — Supporting Information [file ADVS-13-e17704-s001.pdf]

Supplementary Information: The interplay of magnetic order with the electronic scattering and crystal-field effects in a metallic ferromagnet

Payel Shee,[1] Tanaya Halder,[1] Chia-Jung Yang,[2] Nainish Tickoo,[1] Ratiranjan Samal,[1] Ruta Kulkarni,[3] Shishir K. Pandey,[4,5] Vikas Kashid,[6,7] Ashis K. Nandy,[1] Arumugam Thamizhavel,[3] Anamitra Mukherjee,[1] and Shovon Pal[1]

[1]*School of Physical Sciences, National Institute of Science Education and Research, An OCC of HBNI, Jatni, 752 050 Odisha, India*

[2]*Department of Materials, ETH Zurich, 8093 Zurich, Switzerland*

[3]*Department of Condensed Matter Physics and Materials Science, Tata Institute of Fundamental Research, 400 005 Mumbai, India*

[4]*Department of General Sciences (Physics), Birla Institute of Technology and Science, Pilani, Dubai Campus, Dubai International Academic City, 345 055 Dubai, United Arab Emirates*

[5]*Department of Physics, Birla Institute of Technology and Science, Pilani, Hyderabad Campus, 500 078 Telangana, India*

[6]*Department of Physics, Savitribai Phule Pune University, 411 007 Pune, India*

[7]*MIE-SPPU Institute of Higher Education, Doha, Qatar*

(Updated: November 17, 2025)

This supplementary information contains further details on phonon modes in PrSi at THz frequencies, imaginary part of the THz conductivity, localization parameter, Inverse Partic-

ipation Ratio (IPR), the magnetic structure factor, the frequency-integrated spectral weight of the real part of THz conductivity and the CEF line-widths. The contents are sectionized as:

S1. Phonon calculations

S2. Imaginary part of THz conductivity

S3. Localization parameter

s4. Inverse Participation Ratio (IPR)

S5. The structure factor

S6. Frequency-integrated spectral weight

S7. CEF line-widths

S1 Phonon calculations

Density-functional theory (DFT) calculations are performed using projector-augmented wave method [1,2] as implemented within the Vienna *Ab-initio* Simulation Package (VASP) [3]. The generalized-gradient approximation (GGA) with Perdew-Burke-Ernzerhof (PBE) functional form is used for the calculation of exchange-correlation energy [4]. For Pr and Si atoms, $4f^35s^25p^6$ and $3s^23p^2$ are considered as valence configurations, respectively. The full lattice optimization of the orthorhombic primitive crystal structure of PrSi (SG: *Pnma*, # 62) is done with energy and Hellmann-Feynman force convergence criteria of 10^{-7} eV and 10^{-3} eV/Å, respectively. We used a Plane-

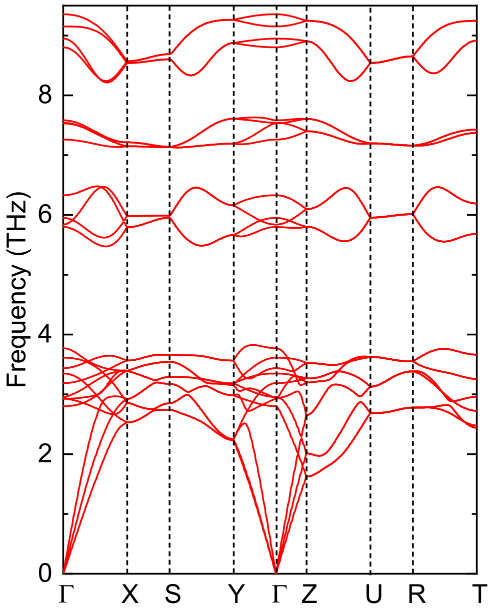

Figure S1: The calculated phonon dispersion using the finite differences method with a $2 \times 2 \times 2$ supercell of PrSi.

Wave cutoff energy of 350 eV and an $11 \times 7 \times 5$ Γ-centered k-mesh for Brillouin zone sampling. A Gaussian smearing width of 0.05 eV was considered in our calculations. We find a small deviation ($< 0.8\%$) in lattice parameters during the optimization of the crystal structure. Phonon dispersion is then calculated using the finite differences method considering a $2 \times 2 \times 2$ supercell. The post-processing of phonon calculations is done using Phonopy software [5,6]. The Infrared (IR) active modes are analyzed using the SAM module [7] of the Bilbao Crystallographic Server.

The calculated phonon dispersion of PrSi is shown in Fig. S1. Consistent with the experimental finding, in our calculations as well, we could not find any unstable phonon modes. The IR active modes for PrSi can be found using the SAM module [7] of the Bilbao Crystallographic Server. There are three IR-active modes allowed corresponding to the 4c Wyckoff positions of Pr and Si,

Table 1: Irreducible representations of IR-active phonon (Irreps) modes at the zone center are listed for PrSi corresponding to the 4c Wyckoff positions.

Frequency	Irreps
2.80 THz	B_{3u}
2.94 THz	B_{1u}
5.74 THz	B_{3u}
5.95 THz	B_{1u}
6.33 THz	B_{2u}
7.26 THz	B_{3u}
7.58 THz	B_{1u}

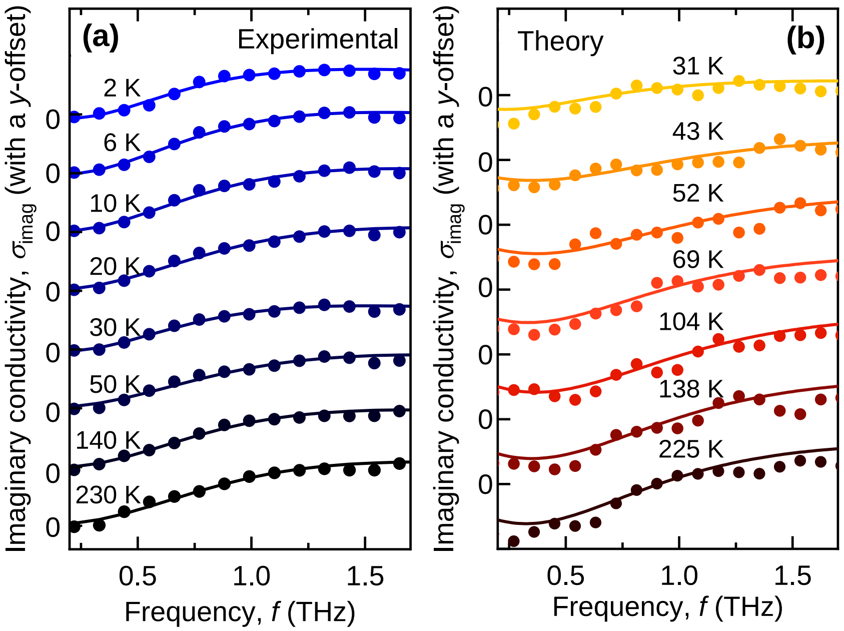

Figure S2: **(a)** Imaginary part of THz conductivity obtained from our experiments. **(b)** Imaginary part of THz conductivity obtained from the classical Kondo-lattice model. The solid lines represent fitted curves using the Drude-Smith model.

irreducible representations for which are B_{1u}, B_{2u}, and B_{2u}. The frequency of these modes is listed in Table 1 at the Γ-point.

S2 Imaginary part of THz conductivity

The temperature-dependent imaginary part of THz conductivity is plotted in Fig. S2a. The solid lines depict the Drude-Smith model as per the imaginary part of Eq.1 of main manuscript. Figure S2b shows the imaginary part of THz conductivity as obtained from the CKLM via the Kramers-Krönig transformation, supporting our experimental data above T_{CK}. In addition, we have fitted the theoretically modeled data using Drude-Smith model, which shows close agreement with the

experimental data.

S3 Localization parameter

Within the Drude-Smith model, the localization parameter provides an information on the degree of confinement (and hence the carrier scattering) in the system. The value of c can range from -1 to 0 where $c = -1$ would correspond to maximum amount of carrier scattering in the system. By modeling the experimentally obtained THz conductivity using the Drude-Smith model, we extract the temperature dependence of the localization parameter (c), which is plotted in Fig. S3. We find that c lies within -1 to -0.75, indicating a higher degree of scattering within the system. In the high temperature region (i.e., $T > T_{\mathrm{C}}$), when the spins are randomly oriented, more carrier scattering prevails leading to c being close to -1. On the other hand, when we are in the low temperature range (i.e., $T < T_{\mathrm{C}}$), the emergence of domains creates an additional degree of confinement in the system. Thus, the localization parameter again assumes a value close to -1. Near the phase transition, however, as the spins start to order, the material experiences a maximum change in its localization environment. Such a change in the electronic environment can potentially act as a seed to the dramatic change in the material's magnetic entropy, eventually leading to a giant magnetocaloric effect in PrSi as reported in Ref. [8]. To bring out this correspondence, we overlay the magnetocaloric data as a function of temperature in Fig. S3.

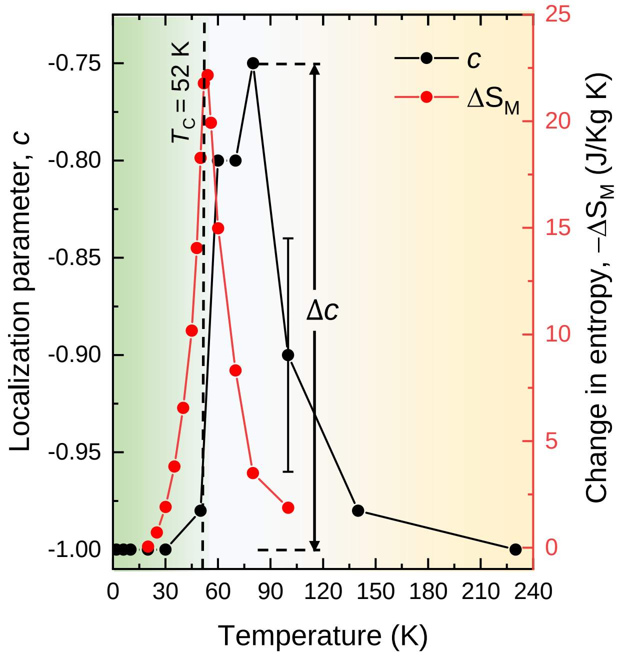

Figure S3: Temperature-dependent localization parameter c extracted from the Drude-Smith (DS) modeling of the THz conductivity (black curve), showing a maximum change of Δc close to the T_C. The temperature-dependent change in the magnetic entropy (adapted with permission from Ref. [8]) is plotted for correspondence. The yellow- and green-shaded regions show the paramagnetic and the ferromagnetic phases, respectively. The vertical dashed-line marks the T_C of the system. The error bar indicates the average standard errors obtained from the Drude-Smith modeling of the experimental data.

S4 Inverse Participation Ratio (IPR)

We calculated the inverse participation ratio (IPR) to quantify the localization properties of the spin channels. We used the standard definition of IPR for spin channel σ as: $\sum_{\mathbf{r}_i} |\psi_{\alpha,\sigma}(\mathbf{r}_i)|^4$, where $\psi_{\alpha,\sigma}(\mathbf{r}_i)$ is the amplitude of the αth eigenstate at position $\mathbf{r}_i$. We choose a small window in

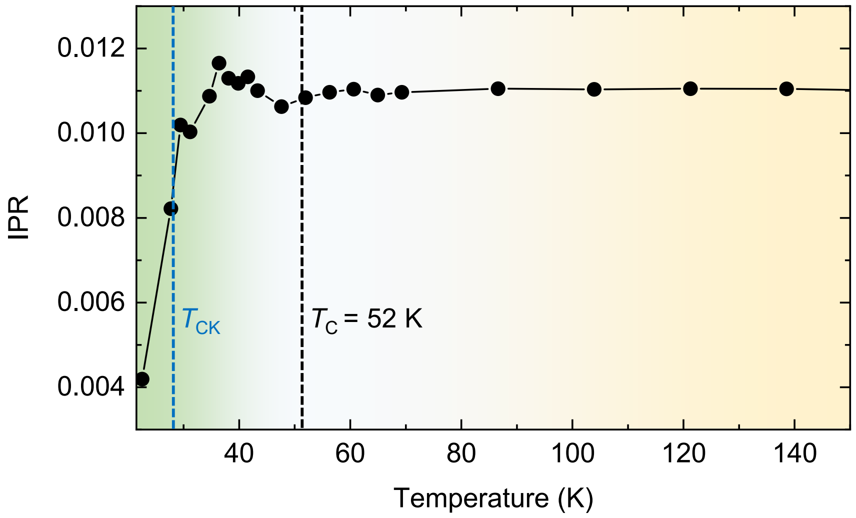

Figure S4: Temperature-dependence inverse participation ratio (IPR). The yellow- and green-shaded regions show the paramagnetic and the ferromagnetic phases, respectively. The vertical black and blue dashed-line marks the T_C and T_{CK} of the system.

energy around the chemical potential and take an average of the IPR over the eigenstates within that window. The resulting IPR is further averaged over 100 Monte-Carlo samples at every temperature and five independent Monte-Carlo runs with different random number seeds. In Fig. S4, we show the temperature dependence of the IPR for the majority spin channel. It is known that IPR scales as inverse square of the localization length scales, which systematically allows us to quantify the increased scattering of the itinerant electrons from the localized moments that become more disordered with increasing temperature. The enhanced scattering thereby reduces the mean free path of the electrons and leads to a suppression of the Drude weight as $\omega \to 0$, consistent with literature [9]. We have also checked that for $T << T_{CK}$, the IPR reduces to $1/L^2$, L being the linear system dimension, where the local moments are aligned in a ferromagnetic order and offer

negligible scattering of the itinerant electrons.

S5 The structure factor

For the characterization of the magnetic ordering in our system, we look at the magnetic structure factor $S(q)$, given by

$$S(q) = \frac{1}{N^2} \sum_{i,j} e^{i\mathbf{q}\cdot(r_i - r_j)} \left\langle \mathbf{S}_i \cdot \mathbf{S}_j \right\rangle,$$

where $\mathbf{q} = 0,0$ is the wave-vector considered, as we are interested in the ferromagnetic order. N is the dimension of the system. $\mathbf{S}_i$ and $\mathbf{S}_j$ are the spins at the $i-$th and $j-$th site, respectively. r_i and r_j give the position of the respective spins. The structure factor gives an estimation for the

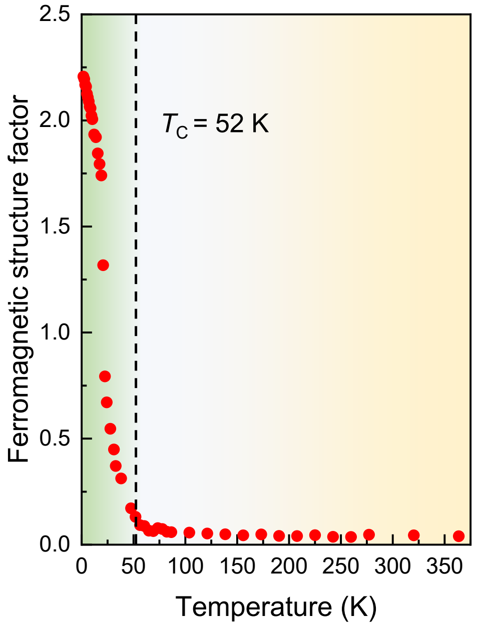

Figure S5: Temperature-dependent ferromagnetic structure factor, showing the onset of magnetic order in the system. The yellow- and green-shaded regions show the paramagnetic and the ferromagnetic phases, respectively. The vertical dashed-line marks the T_C of our system.

ordering temperature, see Fig. S5.

S6 Frequency-integrated spectral weight

To verify our fitting results obtained from the double-Lorentz model, we evaluated the frequency-integrated spectral weight of the corresponding peaks. We know from Kubo's formula,

$$\int \sigma_r(\omega)\, d\omega = \frac{\pi n_{\text{eff}} e^2}{2m^*} \tag{1}$$

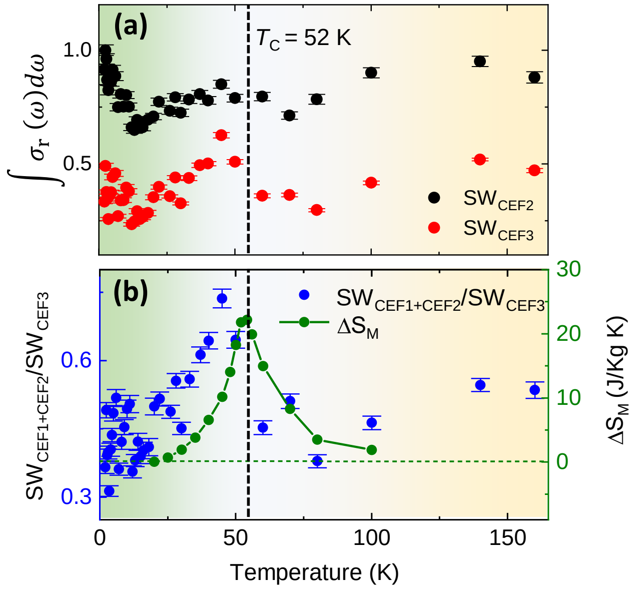

Figure S6: **(a)** Temperature-dependent frequency-integrated spectral weight as per the Kubo's formula. **(b)** Ratio of the spectral weights corresponding to CEF_2 and CEF_3 as a function of temperature. The temperature-dependent change in the magnetic entropy (adapted with permission from Ref. [8]) is plotted for correspondence. The yellow- and the green-shaded regions show the paramagnetic and the ferromagnetic phases, respectively. The vertical dashed-line marks the T_C of our system. Here, the error bars are associated with the standard errors of numerical integration.

where, σ_r is the real part of the THz conductivity, n_{eff} is the effective number of electrons involved in the absorption process, e is the electronic charge, and m^* is the effective mass of the charge carriers [10]. An integration on the CEF peak would give us an idea on the number of carriers involved in the process. In Fig. S6a, we see that the occupation corresponding to CEF_2 transition is always higher at all temperature. We expect that because that is the lower excited state. However, at T_c the occupation corresponding to CEF_3 increases. The relative spectral weight plotted in Fig. S6b as a function of temperature, clearly corroborates our results shown in Fig.4c of the main manuscript.

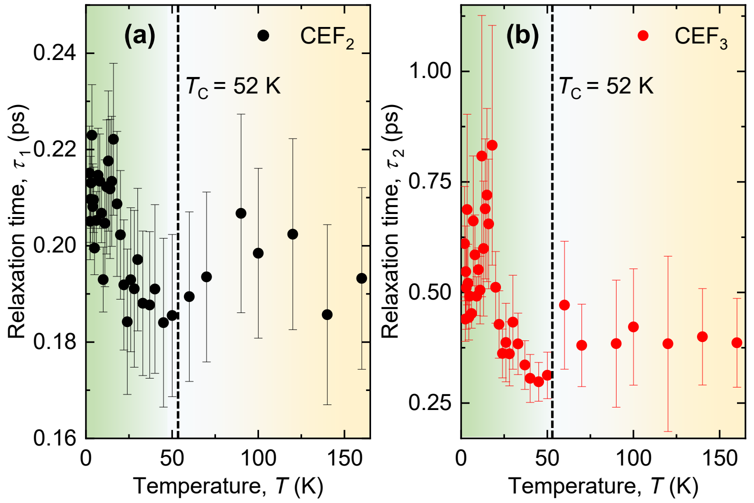

Figure S7: The temperature dependence of the relaxation times obtained from DL modeling, corresponding to **(a)** CEF_2 (i.e., τ_1) and **(b)** CEF_3 (i.e., τ_2). The yellow- and the green-shaded regions show the paramagnetic and the ferromagnetic phases, respectively. The vertical dashed-lines mark the T_C of our system.

S7 CEF line-widths

We observe that the relaxation time corresponding to both the CEF peaks increases as we lower the temperature (see Figs. S7a and S7b). In other words, the linewidth ($1/\tau$) corresponding to the peaks decreases, which not only supports the underlying thermal broadening in the material but also corroborates the onset of the magnetic ordering of the system. This dependence substantiates that our second approach with modeling of the CEF environment using DL oscillators throughout the temperature range. Note that we are not expecting a dramatic increase of the line-widths as increase the temperature since the system is metallic, where the CEF transitions are buried deep below the Fermi level.

References

1. G. Kresse, and D. Joubert, From ultrasoft pseudopotentials to the projector augmented-wave method, Phys. Rev. B **59**, 1758 (1999).

2. P. E. Blöchl, Projector augmented-wave method, Phys. Rev. B **50**, 17953 (1994).

3. G. Kresse, and J. Furthmüller, Efficient iterative schemes for *ab initio* total-energy calculations using a plane-wave basis set, Phys. Rev. B **54**, 11169 (1996).

4. J. P. Perdew, K. Burke, and M. Ernzerhof, Generalized gradient approximation made simple, Phys. Rev. Lett. **77**, 3865 (1996).

5. A. Togo, L. Chaput, T. Tadano, and I. Tanaka, Implementation strategies in phonopy and phono3py, J. Phys.: Condens. Matter **35**, 353001 (2023).

6. A. Togo, First-principles phonon calculations with phonopy and phono3py, J. Phys. Soc. Jpn. **92**, 012001 (2023).

7. E. Kroumova, M. I. Aroyo, J. M. Perez-Mato, A. Kirov, C. Capillas, S. Ivantchev, and H. Wondratschek, Bilbao crystallographic server: Useful databases and tools for phase-transition studies, Phase Transitions **76**, 155 (2003).

8. P. K. Das, A. Bhattacharyya, R. Kulkarni, S. K. Dhar and A. Thamizhavel, Anisotropic magnetic properties and giant magnetocaloric effect of single-crystal PrSi, Phys. Rev. B **89**, 134418 (2014).

9. S. Kumar, and P. Majumdar, Transport and localisation in the presence of strong structural and spin disorder, Eur. Phys. J. B **46**, 237 (2005).

10. D. N. Basov, R. D. Averitt, D. van der Marel, M. Dressel, and K. Haule, Electrodynamics of correlated electron materials, Rev. Mod. Phys. **83**, 471 (2011).
